# Meta-analysis of the effectiveness of exercise as an intervention for suicidal tendency in depressed patients

**DOI:** 10.3389/fpsyg.2025.1517492

**Published:** 2025-06-13

**Authors:** Wenli Wang, Hairong Liu, Qiangming Feng, Yuanming Peng, Yanran Si

**Affiliations:** Faculty for Physical Education, Shanghai International Studies University, Shanghai, China

**Keywords:** depression, exercise intervention, suicidal tendency, meta-analysis, systematic review

## Abstract

**Background:**

The objective of this study is to systematically evaluate the effects of exercise interventions on depressive symptoms and suicidal tendencies in patients with depression, and to investigate the differential impacts of various exercise programs on alleviating depressive symptoms.

**Methods:**

Computerized searches were conducted in PubMed, The Cochrane Library, Embase, WOS, EBSCO, CNKI, Wanfang, and VIP databases from their inception to May 5, 2025. Randomized controlled trials assessing the effect of exercise interventions on suicidal tendencies in depressed patients were screened by two independent researchers. The PEDro scale assessed study quality, and GRADEPro evaluated evidence quality. ReMan 5.4.1 was used for Meta-analysis and publication bias test. Standardized mean difference, Odds Ratio, and 95% CI were used as effect statistics.

**Results:**

A total of 5 papers (5 RCTs with 796 patients) were included in this study. The results showed that exercise reduced depressive symptoms (SMD = −0.99, 95% CI [−1.95, −0.03], *p* = 0.04). But did not prevent suicidal ideation (SMD = −1.49, 95% CI [−4.33, 1.35], *p* = 0.30) and incidents of suicidal ideation in depressed patients (OR = 0.79, 95% CI [0.08, 7.67], *p* = 0.84). Among these, the heterogeneity of outcomes for depression was high, with potential influences including patient age, frequency, duration, and period of exercise, leading to moderate quality of evidence. Subgroup analyses showed that exercise had a antidepressant effect in middle-aged patients (SMD = −0.60, 95% CI [−1.06, −0.14], *p* = 0.01) and was effective from with a period of <12 weeks (SMD = −0.76, 95% CI [−1.05, −0.47], *p* < 0.00001), duration >30 min (SMD = −0.89, 95% CI [−1.32, −0.45], *p* < 0.00001), and frequency ≥3 times/week (SMD = −0.60, 95% CI [−1.06, −0.14], *p* = 0.01) had the largest effect size.

**Conclusion:**

Physical exercise was associated with an improvement in depressive symptoms. In contrast, physical exercise did not show a statistically effect on reducing suicidal tendencies and suicide risk among patients. Through subgroup analysis, it was found that the most beneficial intervention for physical exercise to relieve depressive symptoms was ≥3 times/week, lasting >30 min over <12 weeks, and combining various forms of aerobic exercise. For suicidal tendencies, however, a dose–response relationship could not be established due to limited literature.

**Systematic review registration:**

PROSPERO, Identifier CRD42024568335.

## 1 Introduction

Depression is a major mental disorder. According to the World Health Organization, a total of 280 million people worldwide suffer from depression, and it is expected to rank first in the global burden of disease by 2030 (World Health Organization, 2017). The clinical manifestations of depressed patients are usually low mood, loss of interest and pleasure, and lack of energy or fatigue (Herrman et al., 2019). And depressed patients have a high risk of suicide, and some of them show suicide attempts or behaviors. It has been found that 3 to 8% of depressed patients die by suicide, which is about 20 times higher than the risk of suicide in the general population (Coryell et al., 2019), and about more than 700,000 people among patients lose their lives every year (World Health Organization, 2023). Suicidal tendency as a precursor of suicidal behavior, it is necessary to intervene in time to avoid tragedy (Wilchek-Aviad and Cohen-Louck, 2022).

It has been well documented that exercise is effective in improving depressive symptoms as well as reducing suicidal tendency (Grasdalsmoen et al., 2020), and the two may share some of the neural mechanisms. Studies have shown that physical exercise can change the content of certain neurotransmitters in the body (Norevik et al., 2024), such as dopamine, 5-hydroxytryptamine and other monoamines, as well as endorphins (He et al., 2012), and at the same time activate the prefrontal lobes of the brain at a wider level (Radak et al., 2013), improving the emotional control of depressed patients and enabling individuals to better regulate negative emotions (Lou et al., 2022; Li et al., 2024). Physical exercise has a positive impact on improving mood by preventing the negative effects of stress on health (Mikkelsen et al., 2017; Burtscher and Burtscher, 2024) and improving patients’ depressive symptoms and suicidal tendency (Radak et al., 2013; Repple and Opel, 2021). On the other hand, in addition to depressive symptoms, physical activity attenuates important risk factors associated with suicide, such as anxiety symptoms, sleep disorders, alcohol abuse and overweight (Mikkelsen et al., 2017; Rosenthal et al., 2018; Dinis and Bragança, 2023; Uhaq et al., 2023; Falkai et al., 2022; Wang and Liu, 2025), which are likewise thought to be negatively correlated with the level of physical activity (Bettariga et al., 2024). In addition, exercise has the advantages of maneuverability, high compliance, and few side effects (Heissel et al., 2023), making it a promising intervention (Kvam et al., 2016).

A review of previous literature shows that exercise is effective in improving depressive symptoms and also reduces suicidal tendencies in suicidal individuals. In contrast, the inhibitory effect of exercise on suicidal tendencies in depressed patients, as a high-risk group for suicide, is unclear and lacks a systematic review. While previous systematic reviews have discussed various types of exercise as having a palliative effect on depression (Noetel et al., 2024), the present study further explored the potential for exercise to improve outcomes in suicidal depressed patients. This study intends to systematically evaluate the intervention effect of exercise on Suicidal tendency in depressed patients, to provide a scientific exercise program for them, and to provide a clinical reference.

## 2 Methods

The study was conducted following the PRISMA statement of priority reporting Items for Systematic Reviews and Meta-analysis (Moher et al., 2010) and Cochrane workbook (Cochrane Training, 2010) requirements. This study was registered on PROSPERO under registration number no. CRD42024568335. Registration platform: www.crd.york.ac.uk.

### 2.1 Search strategy

Two researchers independently searched seven databases—Web of Science, Pub Med, The Cochrane Library, Embase, China national knowledge internet (CNKI), Wan Fang Data, and VIP—from their inception until May 5, 2025. The search strategy combined subject headings with free-text terms, finalized after several preliminary searches, and was enhanced by manual checks, including tracing back to references of included studies when necessary. Check the list of references in the included literature and manually look for potentially relevant studies that were not searched in the database. Ensure that all relevant research literature has been taken into account.

#### 2.1.1 Search terms

In this study, the search terms for databases in different languages are specified in the corresponding languages. And according to the different requirements of different databases, corresponding adjustments are made to the subject thesaurus, the use of free words and the syntax of operations. This ensures that the search strategy maximises the coverage of relevant literature in each database and avoids omitting important studies. For example: (exercise OR sports OR athletic OR training OR physical activity OR aerobic exercise OR resistance exercise OR body and mind exercise) AND (depressive disorder OR depressive neuroses OR depressive neurosis OR endogenous depression OR depressive syndrome OR neurotic depression OR melancholia OR unipolar depression OR endogenous depression OR depressive) AND (suicide OR suicidal ideation OR suicidal tendency OR suicidal behavior OR suicide attempt).

### 2.2 Eligibility criteria

Inclusion criteria:

1. Study subjects: ① Age ≥ 18 years old. ② Meet the International Classification of Diseases (ICD) and the Diagnostic and Statistical Manual of Mental Disorders (DSM) diagnostic criteria for depression. ③ Adults diagnosed with depression based on other validated clinical diagnostic criteria. ④ Adults who are suicidal as judged by scale scores or behavior.
2. Interventions: at least one experimental group used an exercise intervention or an exercise intervention based on conventional treatment.
3. Control group: use of pharmacological interventions, psychological interventions, conventional treatments, and exercise interventions that differentiate from the exercise modalities of the experimental group.
4. Outcome indicators: ① depressive symptoms: using Hamilton Depression Scale (HAMD-24, HRSD-17, HAM-D, HAMD, HAM-D17), Geriatric Depression Scale (GDS), Beck Depression Inventory (BDI), Inventory of Depressive Symptomatology-Self Report (IDS-SR), Self-rating Depression Scale (SDS) were used as evaluation tools. ② Suicidal tendency: Beck Scale for Suicide Ideation (BSI); Beck Hopelessness Scale (BHS); Hamilton Depression Scale (HAMD 3: suicide); Self-rating Idea of Suicide Scale (SIOSS). ③ Occurrence of suicidal tendency: suicide attempts, Suicidal tendency, suicide deaths.
5. Type of study: randomized controlled clinical trial.

Exclusion criteria:

① Reviews, conference papers, retrospective studies and systematic evaluations, non-randomized controlled experimental research literature. ② Interventions or intervention subjects did not meet the inclusion criteria. ③ The outcome indicators did not meet the inclusion criteria, the data could not be extracted, or there were obvious errors. ④ Physiotherapy Evidence Database scale (PEDro) (Ludyga et al., 2020) score <4. ⑤ Only one of the duplicate published literature was included. ⑥ For literature lacking original data or where data is unavailable, we will initially contact the corresponding author to reasonably request the original data. If the author cannot be reached or fails to respond, the literature will be excluded from the study.

### 2.3 Data collection process

#### 2.3.1 Selection process

Initially screened articles are imported into Endnote 21 for duplicate removal. The screening is independently performed by two researchers based on the inclusion and exclusion criteria. The process begins with a review of titles and abstracts for preliminary selection, followed by a full-text reading and downloading of articles that meet the criteria. After screening, results are compared, and any discrepancies are discussed with a third researcher to finalize inclusion decisions.

#### 2.3.2 Data items

A standardized protocol is employed to extract pertinent information from the literature. This task is also independently carried out by two researchers for the included articles. For missing or unclear data, direct contact with the original authors via email is made to acquire and verify the information. In cases of conflicting information inclusion, a consensus is reached through discussion with a third researcher. The extracted data encompasses: ① Basic details (author, year, country, age, sample size, postoperative intervention time) ② Experimental specifics (type, duration, frequency) and outcome measures.

### 2.4 Study risk of bias assessment

The quality assessment tool Physical Therapy Evidence Database scale (PEDro) (Ludyga et al., 2020) was used to evaluate the methodological quality of randomized controlled trials. This scale has a total score of 10 points, with 9–10 categorized as high quality studies, 6–8 categorized as higher quality studies, 4–5 categorized as average quality studies, and less than 4 categorized as low quality studies.

Evidence quality evaluated using GRADEPro (Hultcrantz et al., 2017). The evaluation of the quality of evidence for outcome indicators encompasses five downgrading factors: publication bias, inconsistency, imprecision, indirectness, and study limitations. The evidence is classified into four levels based on the degree of downgrading: very low, low, moderate, and high. Specifically, a three-level downgrade results in very low evidence, a two-level downgrade results in low evidence, a one-level downgrade results in moderate evidence, and no downgrade results in high evidence. The quality assessment is independently conducted by two researchers. In cases of disagreement, a third researcher is consulted to reach a consensus through discussion.

### 2.5 Synthesis methods

The software used for data analysis was Review Manager 5.4. Heterogeneity was evaluated using the *p*-value and *I*^2^ statistic. If significant heterogeneity was detected (*I*^2^ ≥ 50%, *p* < 0.10), a random effects model was applied; otherwise, a fixed effects model was used. The SMD (Standardized Mean Difference) and OR (Odds Ratio) were calculated, along with a 95% confidence interval. We calculated the standardized mean difference (SMD) using the mean and standard deviation (SD) from the pre-test (baseline) and post-test (post-intervention). The formula used is as follows: $SMD=\frac{M_{1}-M_{2}}{\sqrt{\frac{\left(SD_{1}^{2}+SD_{2}^{2}\right)}{2}}}$ . Where M_1_ and M_2_ represent the means of the experimental group and the control group, respectively; SD_1_ and SD_2_ represent the standard deviations of the experimental group and the control group, respectively. The change in standard deviation (i.e., the change in standard deviation from baseline to endpoint) was estimated by the following equation: SD_change_ = $\sqrt{SD_{baseline}^{2}+SD_{endpoint}^{2}-2\times \mathrm{Correlation}\times SD_{baseline}\times SD_{endpoint}}$ . Where: SD_baseline_ is the standard deviation of the baseline; SD_endpoint_ is the standard deviation of the endpoint. Correlation is the correlation between the baseline and the endpoint, with a default assumption of 0.5 (Walters et al., 2019; Nasser, 2020). Sensitivity analysis involved sequentially excluding individual studies. If heterogeneity was substantial, a descriptive analysis was performed. The Egger’s test was used to test for publication bias.

## 3 Results

### 3.1 Study selection

**Figure 1:**
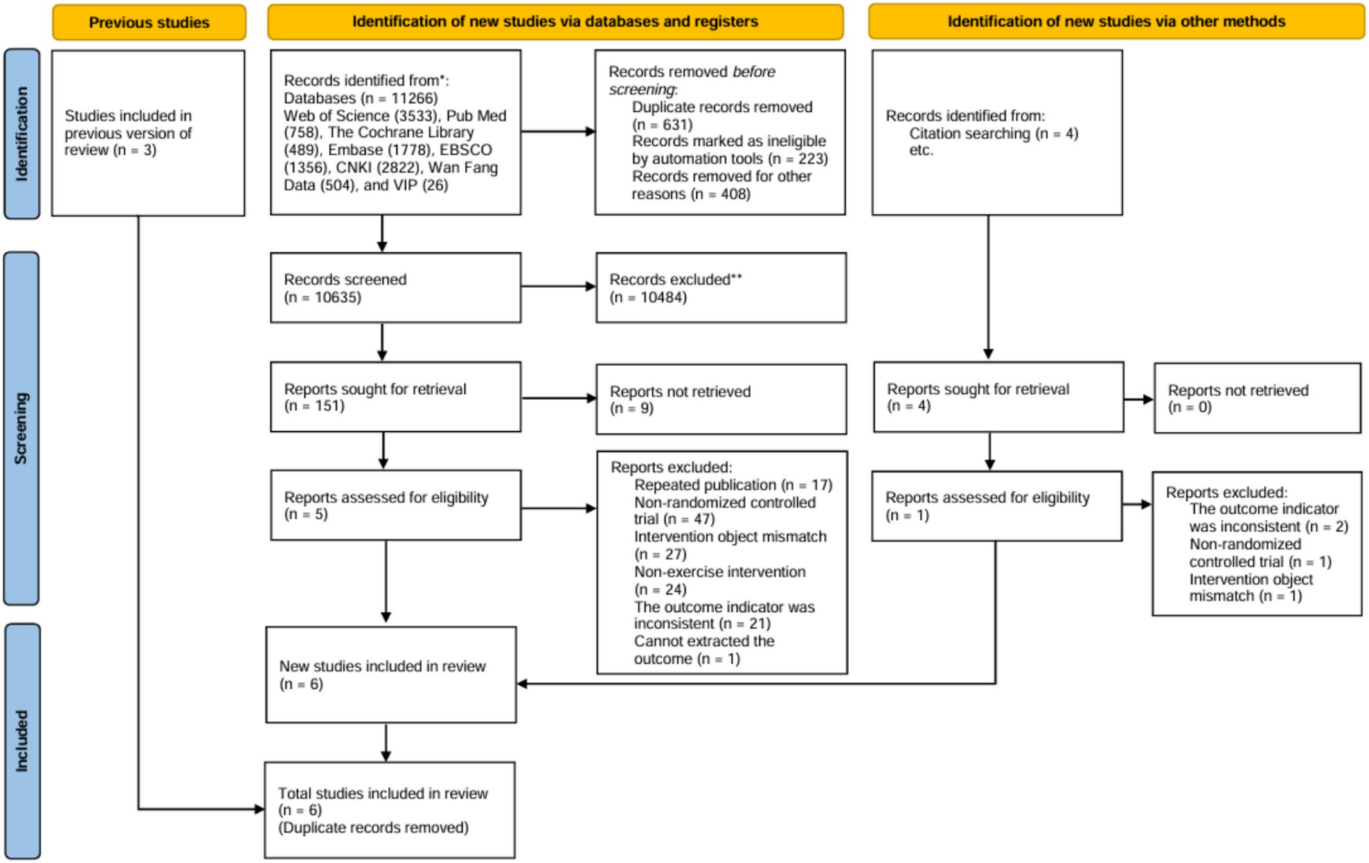
Literature screening flow chart.

A systematic online search using computers retrieved 11,266 articles, with an additional 1 articles were found through manual search methods. Retrieved libraries include: Web of Science (3533), Pub Med (758), The Cochrane Library (489), Embase (1778), EBSCO (1356), China national knowledge internet (CNKI) (2822), Wan Fang Data (504), and VIP (26). After removing duplicates, 10,635 articles remained. These were initially screened by examining titles and abstracts, followed by a thorough review of the full texts to exclude those that did not fulfill the inclusion criteria. Consequently, 6 articles were selected for inclusion in the analysis, as illustrated in Figure 1.

### 3.2 Study characteristics

The 6 included papers were published between 2012 and 2022 and included a total of 796 patients (399 in the experimental group and 397 in the control group). Subjects were predominantly patients with major depression, with some of the literature including mild and moderate patients. Exercises involved in the intervention included many forms of aerobic exercise, with a cycle range of 5 to 12 weeks, a frequency of 1 to 5 times per week, a single session duration of 30 to 180 min (see Table 1).

**Table 1:** Basic features of included studies.

Study	Nation	Diagnostic tools (sample source)	Depression level	Disease duration	Sample size		Age (years)	Gender (M/F)	Intervention measure		Outcome index	
					T	C			T	C	Continuous variables	Dichotomous variables
Yan et al. (2020)	China	Doctor’s Diagnosis (Hospital)	Severe	7.9 ± 3.0 8.6 ± 3.8	50	50	42.3 ± 5.9 43.1 ± 6.0	21/29 19/31	Aerobic exercise (5 times/week for 5 weeks, 30 min/time)	Conventional Therapy	HAMD-24(↓)	Incidence of suicide deaths (—)
Sturm et al. (2012)	Austria	BDI (Hospital)	Severe		10	10	45.1 ± 10.4 41.0 ± 6.3	3/7 3/7	Hiking (3 times/week for 9 weeks, 120-180 min/time, 65–75% Hrmax)	Conventional Therapy	BDI(↓) BSI(—) BHS(↓)	
Haussleiter et al. (2020)	Germany	DSM-IV/SCID-I/SCID-II/HAMD (Hospital)	Moderate–Severe	7.0 ± 6.3 11.67 ± 9.03	36	40	43.94 ± 13.24 46.43 ± 11.60	5/31 6/32	Standardized exercise (3times/week for 6 weeks, 50 min/time)	Conventional Therapy	HAMD(↓) HAMD 3(↓)	
Krogh et al. (2012)	Denmark	DSM-IV (Hospital)	Severe		53	47	43.4 ± 11.2 39.7 ± 11.3	22/37 16/40	Aerobic exercise (3 times/week for 12 weeks, 30 min/time)	Stretching	HAMD-17(—) BDI(—)	Number of suicidal tendency deaths (—)
Kong (2022)	China	SDS	Mild–Severe		250	250	College students	—	dance (1 times/week for 12 weeks)	Conventional Therapy	SDS(↓) SIOSS(↓)	

DSM-IV/DSM-IV-TR/DSM-5: American Diagnostic and Statistical Manual of Mental Disorders (4th/5th editions); ICD-10: International Classification of Diseases, 10th edition; HAMD/HAMD-24/HAMD-17: Hamilton-Rating Depression Scale (17th/24th editions); SCID-I/SCID-II: Personality Disorder Diagnostic Inventory (1st edition/2nd edition); BDI/BDI-II: Beck Depression Self-Rating Inventory (1st edition/2nd edition); SDS: Self-Report Depression Scale; BSI: Beck Suicidal Ideation Inventory; BHS: Beck Hopelessness Scale; HAMD 3: suicide; SIOSS: Self-Inventory of Suicide Scale; T: experimental group; C: control group; M: male; F: female. ↓: Significant decrease in the number of scores/occurrences; —: results not significant.

### 3.3 Risk of bias in studies

The quality evaluation tool Physical Therapy Evidence Database (PEDro) scale (Ludyga et al., 2020) was used to evaluate the methodological quality of randomized controlled trials. Of the 5 papers included, the PEDro score ranged from 5 to 8, with a mean of 6. All studies reported between-group statistical analyses, point measures, and difference-in-difference scales. Four studies reported randomized grouping methods, including lotteries and computerized allocation, and one paper (Kong, 2022) referred only to randomization; three studies performed allocation concealment; four papers provided baseline similarity; no study achieved blinding of the participants; one study achieved low implementation bias; three studies provided more than 85% of subject of outcome information; and four studies performed intention-to-treat analysis (Table 2).

**Table 2:** Results of the methodological quality assessment of the included literature.

Study	Random sequence generation	Allocation concealment	Similar baseline	Blinding of participants	Blinding of therapist	Blinding of outcome assessment	Participation rate >85%	Intent-to-treat information	Between group statistical outcome analysis	Point measures and measures of variance	Total score
Yan et al. (2020)	0	0	1	0	0	0	1	1	1	1	5
Sturm et al. (2012)	1	1	1	0	0	0	1	0	1	1	6
Haussleiter et al. (2020)	1	1	1	0	0	0	0	1	1	1	6
Kong (2022)	1	0	0	0	0	0	1	1	1	1	5
Krogh et al. (2012)	1	1	1	1	1	0	0	1	1	1	8

### 3.4 Results of syntheses

The results of this study only include articles that simultaneously focus on depression and suicidal tendencies.

#### 3.4.1 The role of exercise interventions on depressive symptoms

**Figure 2:**
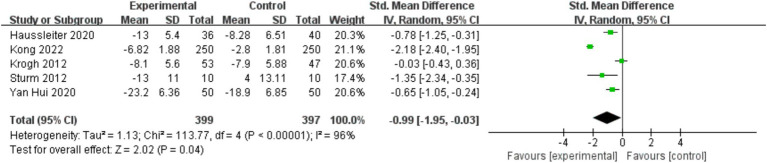
Forest plot of the effect of physical exercise on depressive symptoms in patients.

Five studies reported depression degree symptoms, with a total of 796 patients, with a large heterogeneity among studies ( $I^{2}$ =96%, *p* < 0.00001), which were analyzed using a random-effects model. The results showed that patients in the experimental group had significantly lower depression degree symptoms than those in the control group, and the difference was statistically significant (SMD = −0.99, 95% CI [−1.95, −0.03], *p* = 0.04) (Figure 2).

To investigate whether the heterogeneity among studies was caused by individual studies, sensitivity analyses were performed on studies with high heterogeneity of exercise interventions for depressive symptoms and suicidal tendency in depressed patients, and the combined effects were analyzed by excluding individual studies one by one (Table 3).

**Table 3:** The pooled effect size of depressive symptoms after excluding individual studies.

Outcome index	Study	SMD	95%CI	*p*-value	$I^{2}/\%$
Depression	Yan et al. (2020)	−1.08	−2.23 ~ 0.14	0.08	97
	Kong (2022)	−0.60	−1.06 ~ −0.14	0.01	70
	Sturm et al. (2012)	−0.92	−2.01 ~ 0.18	0.10	97
	Haussleiter et al. (2020)	−1.05	−2.23 ~ 0.14	0.08	97
	Krogh et al. (2012)	−1.24	−2.18 ~ −0.30	0.01	95

The pooled effect size of exercise interventions for depression level symptoms in depressed patients from all studies included in Table 3 was SMD = −0.99, with a 95% CI [−1.95, −0.03] and *p* = 0.04, $I^{2}$ =96%; after excluding Kong (2022), the pooled effect size was SMD = −0.60, with a 95% CI [−1.06, −0.14] and *p* = 0.01, $I^{2}$ =70%, heterogeneity was significantly reduced. The range of SMD after excluding other single studies was −0.92 to −1.24, $I^{2}$ was 97 to 95%, and *p* value ranged from 0.01 to 0.10. Comparison of basic information in the literature revealed that the included population in the literature (Kong, 2022) had a lowest frequency of exercise. The study was excluded because of clinical heterogeneity and a random effects model was used.

#### 3.4.2 The role of exercise interventions on suicidal tendency

**Figure 3:**
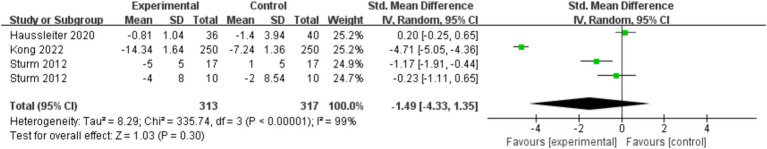
Meta-analysis of exercise intervention for suicidal tendency in depressed patients.

Three papers reported the results of studies on suicidal tendency, with a total of 630 patients, with significant heterogeneity between studies ( $I^{2}$ =99%, *p* < 0.00001), which were analyzed using a random-effects model. The results showed that patients in the experimental group had a significant reduction in suicidal tendency, and the difference was not statistically significant (SMD = −1.49, 95% CI [−4.33, 1.35], *p* = 0.30) (Figure 3).

#### 3.4.3 The role of exercise interventions on the incidence of suicidal tendency

**Figure 4:**
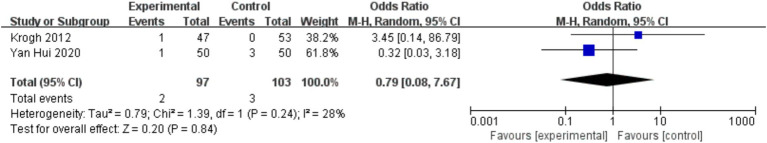
Meta-analysis of exercise intervention for the incidence of suicidal tendency in depressed patients.

Two papers reported the results of adverse events (occurrence of suicidal tendency events) in a total of 200 patients, with no significant heterogeneity between studies ( $I^{2}$ =28%, *p* = 0.24), which were analyzed using a random-effects model. The results showed that the occurrence of adverse events was significantly lower in the experimental group of patients than in the control group, but the difference was not statistically significant (OR = 0.79, 95% CI [0.08, 7.67], *p* = 0.84) (Figure 4).

#### 3.4.4 Analysis of subgroup moderating effects

The effect of exercise on depressive symptoms in depressed patients may be conditioned by different exercise frequency, period, duration, intensity and intervention types. Subgroup moderated effect analyses were performed on the included literature. Because the studies included in this study that provided heart rate or maximum oxygen uptake were of moderate exercise intensity, exercise intensity was no longer grouped. Since all exercise modalities included in the study are aerobic exercises, no further grouping based on exercise type is performed. The subgroups were set up according to the three moderating variables of exercise frequency, period, and duration. The included studies were grouped according to the period of exercise, which was categorized into 2 subgroups: <12 weeks, =12 weeks; the exercise duration was categorized into 30 min, and >30 min; the frequency of exercise was categorized into ≥3 times/week.

The results of the subgroup analysis (Table 4) indicate that all three moderator variables exhibited statistically significant subgroup effects. In terms of heterogeneity sources, the heterogeneity of the exercise duration subgroup decreased to as low as 3%, the exercise period subgroup to 0%, and the exercise frequency subgroup to 70%. Compared to the overall pooled effect (*I*^2^ = 96%), these findings suggest a substantial change in heterogeneity. Therefore, exercise period, duration, and frequency are considered sources of heterogeneity.

**Table 4:** Results of moderating effects for stratified subgroups.

Study characteristics		Groups	Depression level						
			Studies	Sample size	SMD	95%CI	*p*-value	$I^{2}$ /%	*P* (Heterogeneity)
Basic Information	Age	Middle	4	296	−0.60	−1.06 ~ −0.14	0.01	70	0.02
Exercise Elements	Period	<12 weeks	3	196	−0.76	−1.05 ~ −0.47	<0.00001	0	0.44
		12 weeks	2	600	−1.11	−3.21 ~ 0.99	<0.00001	99	0.30
	Duration	=30 min	2	200	−0.34	−0.94 ~ 0.26	0.27	78	0.03
		>30 min	2	96	−0.89	−1.32 ~ −0.45	<0.00001	3	0.31
	Frequency	≥3 times/week	4	296	−0.60	−1.06 ~ −0.14	0.01	70	0.02
Total			5	796	−0.99	−1.95 ~ −0.03	0.04	96	<0.00001

Regarding effect size, a weekly exercise frequency of ≥3 times (SMD = −0.60), an exercise duration of >30 min per session (SMD = −0.89), and an intervention period of <12 weeks (SMD = −0.76) demonstrated a greater effect on alleviating depression in patients, with more active symptom improvement and more pronounced effects.

The population included in this study was primarily middle-aged individuals (30–50 years old), with two studies focusing on university students. After subgrouping by age, the heterogeneity in the middle-aged group decreased to 70%, suggesting that participant age is also a potential source of heterogeneity.

### 3.5 Publication bias assessment

Publication bias was evaluated for outcome indicators with the number of included studies was 5. According to the results of Egger’s test, there was no publication bias in depressive symptoms (*Z* = 0.14, *p* = 0.891), suicidal tendency (*Z* = 1.03, *p* = 0.302), or incidence of suicidal tendency (*Z* = −1.18, *p* = 0.239).

### 3.6 Certainty of evidence

The certainty of outcomes was assessed using the GRADE system and the Guideline Development Tool (GRADEpro GDT) in conjunction with the guidelines provided in Chapter 14 of the Cochrane Handbook for the Systematic Evaluation of Interventions (Cochrane Training, 2010; Gao et al., 2021). The results showed that a total of three outcome indicators were included in the study and all were moderate to high quality outcome indicators. The results of the GRADE quality assessment of the evidence from the included studies are shown in the Table 5.

**Table 5:** Results of the GRADE quality assessment of evidence for endpoints.

Outcomes	Illustrative comparative risks* (95% CI)	Relative effect (95% CI)	No of Participants (studies)	Quality of the evidence (GRADE)	Comments
Depressive Symptoms	The mean depressive symptoms in the intervention groups was0.99 standard deviations lower (1.95 to 0.03 lower)	SMD −0.99 (−1.95 to −0.03)	796 (5 studies)	⊕ ⊕ ⊕⊝ moderate	Lower is better
Suicidal Tendency	The mean suicidal tendency in the intervention groups was1.49 standard deviations lower (4.33 to 1.35 lower)	SMD −1.49 (−4.33 to 1.35)	630 (3 studies)	⊕ ⊕ ⊕⊝ moderate	Lower is better
Occurrence of suicidal tendency events	The occurrence of suicidal tendency in the intervention groups was 6 lower per 1,000 (27 to 158 lower)	OR 0.79 (0.08 to 7.67)	200 (2 studies)	⊕ ⊕ ⊕⊕ high	Lower occurrence in experimental group

## 4 Discussion

This study further confirms that exercise improves depressive symptoms in depressed populations. Other Meta-analyses on depressed patients have similarly confirmed the positive effect of exercise on antidepressants. Heissel et al. conducted a Meta-analysis of the results of 41 studies including 2,264 patients with major depressive disorder, confirming that exercise has a positive effect on decreasing the level of depression in patients with depression (Heissel et al., 2023). Schuch et al. conducted a meta-analysis based on 25 studies (including 9 studies of patients with major depression) with a total of 1,487 participants and found that physical activity had a significant antidepressant effect, especially for patients with major depression (Kvam et al., 2016). Studies with a total of 1,487 participants, a meta-analysis found that physical activity had a significant antidepressant effect, especially in patients with major depression (Schuch et al., 2016).

This study further found that exercise interventions with a frequency of ≥3 times per week, a period of <12 weeks, and a duration lasting >30 min yielded better outcomes. Other related studies have also confirmed the dose–response effect of this intervention regimen. Sousa et al. found that moderate-intensity exercise 3 times per week for 60 min was significantly effective in antidepressant (De Sousa et al., 2021). Li et al. analyzed 35 studies with a total of 5,393 subjects and found that exercise 3 times per week for 40–50 min had the best antidepressant effect (Li et al., 2023). Li et al. reduced patients’ plasma levels of 5-HT, NE, and cortisol through 8-week, 5-times-a-week, 50-min medium-intensity exercise as an intervention, which effectively enhanced the mental health of depressed patients and alleviated depression (Li et al., 2009). Paolucci showed that moderate-intensity exercise lasting 6 weeks reduced TNF-a levels in peripheral blood, which led to improvement of patients’ depressive symptoms (Paolucci et al., 2018).

Many current studies have found that exercise improves suicidal tendencies in depressed patients, but the results based on meta-analyses of current experimental sample sizes are not significant enough. The positive effect of exercise on suicidal tendency has been confirmed by some studies: a meta-analysis by Vancampfort et al. of eight studies with 80,856 participants found that those who were more physically active were less likely to suffer from SI (suicidal ideation) than those who were “inactive,” and that physical activity participation had a negative effect on suicidal tendency (Vancampfort et al., 2018). Taliaferro et al. studied sports participation and suicidal behavior in 450 patients and found that there was an inverse relationship between the two, and that depression and self-esteem fully mediated the relationship between physical activity and suicidal tendency (Taliaferro et al., 2010). Lei Qianle et al. found that physical activity had a significant negative predictive effect on suicidal tendency through a survey of 1,213 people, and the higher the level of physical activity, the brain promotes the production of pleasurable emotions through the secretion of a large amount of endorphins, which reduces the risk of suicide (Lei et al., 2021).

The present study found no significant effect of exercise on reducing adverse suicidal events, and although the small number of available RCTs have failed to draw significant conclusions, some studies continue to suggest that exercise may have unique potential in addressing suicide risk through specific neurobiological mechanisms. Exercise helps to modulate neurotransmitter systems associated with mood regulation, such as serotonin, norepinephrine, and dopamine, which are often dysregulated, particularly in suicide risk populations (Rethorst et al., 2009). In addition, exercise alleviates the negative emotions associated with suicidal ideation by increasing the release of endorphins. Studies have also shown that exercise reduces levels of pro-inflammatory factors (e.g., TNF-*α* and IL-6), which are strongly associated with suicidal tendencies and depression (Dowlati et al., 2010). By reducing inflammation, exercise helps to restore brain function and reduce the risk of suicide. Although these mechanisms provide possible physiological explanations for improved suicidal tendencies, the complexity of suicidal risk calls for future research to further clarify the most effective exercise regimen.

Although the present study provides a basis for exercise in patients with suicidal tendency depressives and provides relevant recommendations on exercise program design, there is still a lack of standardized and more consistent exercise prescription for patients with depression, and the study is still unable to determine which design option is more effective in reducing patients’ suicidal tendency due to the small number of corresponding studies. In addition, the subgroup analyses in this paper only calculated independent elemental effect sizes and did not explore the effects of different combinations of exercise elements. Therefore, the elements and combinations of exercise that influence depressive symptoms and suicidal tendencies in depressed patients could be explored in the future and combined with other outcome indicators and different populations to provide more actionable recommendations for exercise prescription development.

## 5 Conclusion

The results of the present study suggest that although exercise interventions have a significant effect on alleviating depressive symptoms, they have limited effect in improving suicidal tendencies in patients, and there is no evidence to support their ability to prevent suicidal events. Current evidence suggests that exercise is more appropriate for improving depressive symptoms such as low mood, particularly in middle-aged populations. Relatively effective intervention programmes are: ≥3 times/week, >30 min, duration <12 weeks, and using a combination of exercise forms.

Although the present study primarily found limited effects of exercise on suicidal tendency interventions, this highlights the shortcomings of current research in this area. By systematically integrating the current limited evidence, the present study provides a basic reference for further mechanistic research and intervention design in this direction, and lays a preliminary theoretical basis for promoting the application of individualised and systematic exercise prescription in psychological crisis intervention.

## Data Availability

The original contributions presented in the study are included in the article/supplementary material, further inquiries can be directed to the corresponding author.

## Appendix

**Table A1:** Excluded literature: met inclusion criteria but did not provide sufficient data.

Study	Nation	Diagnostic tools	Sample size		Age (years)	Gender (M/F)	Intervention measure		Outcome index	Exclusion of cause
			T	C			T	C		
Bahamin et al. (2012)	Iran	DSM IV	12	12	28.88 ± 36.984	16/8	Hardiness training (10 times, 60 min/ times)	Conventional Therapy	BSSI	No raw data
